# Evaluation of finger strength and spasticity in hemiplegic patients using hand-finger robotic device: A validity and reliability study

**DOI:** 10.1097/MD.0000000000036479

**Published:** 2023-12-08

**Authors:** Sevda Adar, Ali Demircan, Ali İzzet Akçin, Ümit Dündar, Hasan Toktaş, Hilal Yeşil, Selma Eroğlu, Nuran Eyvaz, Ersin Beştaş, Cansu Köseoğlu Toksoy

**Affiliations:** a Department of Physical Medicine and Rehabilitation, Afyonkarahisar Health Sciences University, Afyonkarahisar, Turkey; b Ataturk Vocational School of Health Services, Afyonkarahisar Health Sciences University, Afyonkarahisar, Turkey; c Department of Neurology, Faculty of Medicine, Afyonkarahisar Health Sciences University, Afyonkarahisar, Turkey.

**Keywords:** hemiplegia, robotics, spasticity, strength

## Abstract

We aimed to investigate the validity, reliability, and clinical relevance of Amadeo hand-finger robotic rehabilitation system measurements for evaluating spasticity and strength in hemiplegic patients. In total, 161 participants (107 hemiplegic patients and 54 sex- and age-matched healthy controls) were included in this study. Spasticity was evaluated using the Modified Ashworth Scale, hand motor functions were evaluated using the Fugl-Meyer Assessment Hand Subscale, and hand grip and pinch strength were evaluated using the Jamar hand grip and pinch dynamometer. The Amadeo (Tyromotion) hand-finger robotic rehabilitation system was used to evaluate finger spasticity and strength of the participants. A statistically significant difference was found between the median values of the Modified Ashworth Scale (both clinical and robotic evaluation results) and the mean values of hand flexor and extensor strength measured with the robotic device in patients compared to healthy subjects (*P* < .01). Statistically, excellent agreement was obtained between the clinical and robotic test-retest results of the scale (*P* < .01) (intra-class correlation coefficient, ICC = .98–.99; ICC = .98–.99, respectively). There was a statistically significant positive correlation between the clinical and robotic device results of the Modified Ashworth Scale (*r* = .72; *P* < .01). There was a statistically significant positive correlation between the hand strength values measured with the robotic device, Jamar grip, pinch, and Fugl-Meyer Assessment Hand Subscale scores (*P* < .01) in the patient group. Hand finger spasticity and strength measurements of the Amadeo hand-finger robotic rehabilitation system were valid, reliable, and clinically correlated in stroke patients.

## 1. Introduction

Stroke is the second leading cause of disability and death worldwide.^[1]^ Spasticity is a common complication experienced by 19% to 43% of stroke survivors.^[2]^ The high prevalence of spasticity and limited effectiveness of existing treatment options highlight its significance as a major health problem in neurological rehabilitation.^[3]^

Spasticity management relies on clinical patient assessment.^[4]^ A validated scoring system should be used in the clinical evaluation of spasticity to ensure quantification. The Ashworth Scale (AS) or Modified Ashworth Scale (MAS) are the most frequently employed scales, which measure passive resistance at the joint as perceived by the examiner^[4]^ hence, giving rise to qualitative and subjective data.^[5]^ Most of these scales have major limitations in terms of reliability and inter-rater reproducibility.^[6]^ Owing to differences in clinical background and training, the assessment of spasticity may vary among clinicians, and the evaluation process can also depend on various subtle factors.^[7]^ Furthermore, the time-consuming nature of these assessments may discourage their frequent use in monitoring and evaluating motor recovery.^[7]^

Robot-aided therapy devices are equipped with sensors and built-in technology that enable the automatic measurement of motion kinematics and kinetics, providing an opportunity to assess the patient’s condition.^[8]^ Substantial evidence has shown that robotic systems can be beneficial in rehabilitating people with disabilities. Furthermore, these robotic devices can be used to evaluate upper extremity function during treatment. Spasticity can also be integrated into robotic assessment protocols by considering its effect on the speed and direction of movement of the upper extremity.^[9]^ Robotic devices are thought to have advantages, such as being monitored in every rehabilitation session (even during each movement), emphasizing even small changes, early detection of the healing plateau, and reduction of evaluation bias for spasticity assessment.^[10]^ Although many studies have utilized robotic and other objective measures, their reliability and validity still need to be established to the same extent as clinical scales. Moreover, the relationship between robotic measurements and clinical scales remains largely unknown.^[11]^ To the best of our knowledge, only one study in the literature has evaluated finger spasticity using a hand-finger robot. In this study, it was concluded that the strength measurements correlated with the clinical results, but the test-retest reliability of the spasticity measurements was weak.^[12]^

In this study, we aimed to investigate the validity, reliability, and clinical relevance of Amadeo hand-finger robotic rehabilitation system measurements for evaluating spasticity and strength in our stroke patient population.

## 2. Methods

### 2.1. Participants

**Figure 1.**
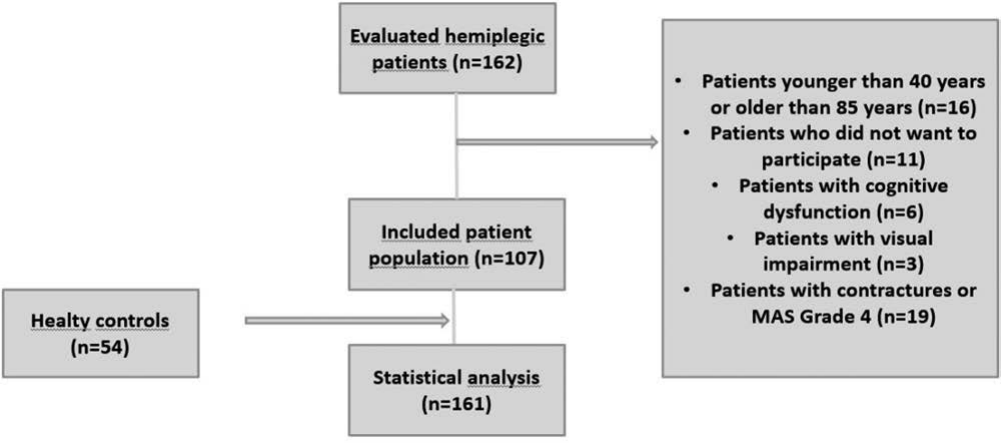
Diagram of patient sample selection and exclusions (boxes on the right).

Patients aged 40 to 85 years, with a history of stroke at least before 6 weeks, who were admitted to the Afyonkarahisar Health Sciences University, Department of Physical Medicine and Rehabilitation between February 15, 2022, and February 15, 2023, were included. The exclusion criteria were an open wound on the hand, acute orthopedic injury, visual impairment that prevents seeing the screen, advanced cognitive impairment, contracture in the wrist and/or fingers, or grade 4 spasticity in finger flexors according to MAS staging. Among these, 107 stroke patients who met the inclusion criteria were included (Fig. 1). The control group included 54 participants, who were matched for age and sex and agreed to participate in the study.

### 2.2. Ethics approval and consent to participate

The study was approved on April 1, 2022 (number 2022/210) by the Afyonkarahisar Health Sciences University, Clinical Research Ethics Committee. The study was also registered at ClinicalTrials.gov (identifier number NCT05662878). All participants provided written informed consent in accordance with the principles outlined in the Declaration of Helsinki.

### 2.3. Clinical assessments

#### 2.3.1. Modified Ashworth Scale (MAS).

An investigator blinded to other clinical measures and robotic assessments performed the clinical assessment of spasticity twice daily at different times using MAS. MAS is one of the most widely used scales in the clinical field because it is easy to apply, does not require special equipment, and can be quickly applied.^[13]^ During the assessment of spasticity, the patients were in a sitting and relaxed position, and the examiner moved the joint at 3 different speeds: V1 (slow), V2 (speed of gravity), and V3 (as fast as possible) to be comparable to robotic device measurements and assigned a rating of resistance on a scale of 0 to 4 (0 = no increased resistance; 1 = slightly increased resistance (catch followed by relaxation or minimal resistance at the end of the range of motion); 1+ = slightly increased resistance (catch followed by minimal resistance throughout less than half of the range of motion); 2 = clear resistance throughout most of the range of motion; 3 = strong resistance; passive movement is difficult; 4 = rigid flexion or extension).^[6,14]^ For the statistical analysis of MAS values, 1 + values were recorded as 2, 2, 3, and 3 as 4.

#### 2.3.2. Fugl-Meyer assessment for upper extremity hand rating subscale (FMA-Hand).

The motor functions of the patients were evaluated clinically using the Fugl-Meyer Assessment for the Upper Extremity (FMA-UE). The Fugl-Meyer Assessment of Upper Extremity (FMA-UE) is a well-established and widely used scale developed to assess and quantify the extent of post-stroke sensorimotor recovery in the upper extremity. It is considered a valid and reliable tool.^[15]^ The FMA-UE scale includes 7 types of hand assessment movements, including grip, pinch, and coordination of fingers (FMA-hand). Each movement was assigned a qualitative rating with a score of 0, 1, or 2, depending on how well it was performed.^[16]^ In this study, we evaluated the hand functions of the participants using the 7-item FMA-Hand subscale.

#### 2.3.3. Jamar dynamometer.

Hand grip strength was measured clinically using a Jamar dynamometer. Finger lateral pinch strength was evaluated using a Jamar digital pinch meter. Measurements were made with the shoulder adjacent to the trunk in adduction and neutral rotation, the elbow in 90° of flexion, the wrist in 0–30° of dorsiflexion, and the ulna in 0°–15° of ulnar deviation with the thumbs up. The patients were asked to squeeze with maximum force, 3 measurements were taken, and the averages were recorded.

### 2.4. Robotic assessment of finger spasticity and strength

Robotic Assessments were performed by a researcher experienced in robotic rehabilitation, trained in using the device, and blinded to the patient’s clinical findings and previous evaluation results. The Amadeo (Tyromotion, Graz, Austria) hand-finger robotic rehabilitation system was used for robotic evaluation of the participants. This is an end-effector device that helps one or all 5 fingers flex and extend independently in a horizontal plane. The robot is not affected by the anatomical limitations of joint alignment owing to its degrees of freedom, and it measures the multi-joint movements of the fingers. The sensors transmitted simultaneous movement information to a computer screen during rehabilitation. Synchronous finger movements are shown in 5 columns.^[17]^

**Figure 2.**
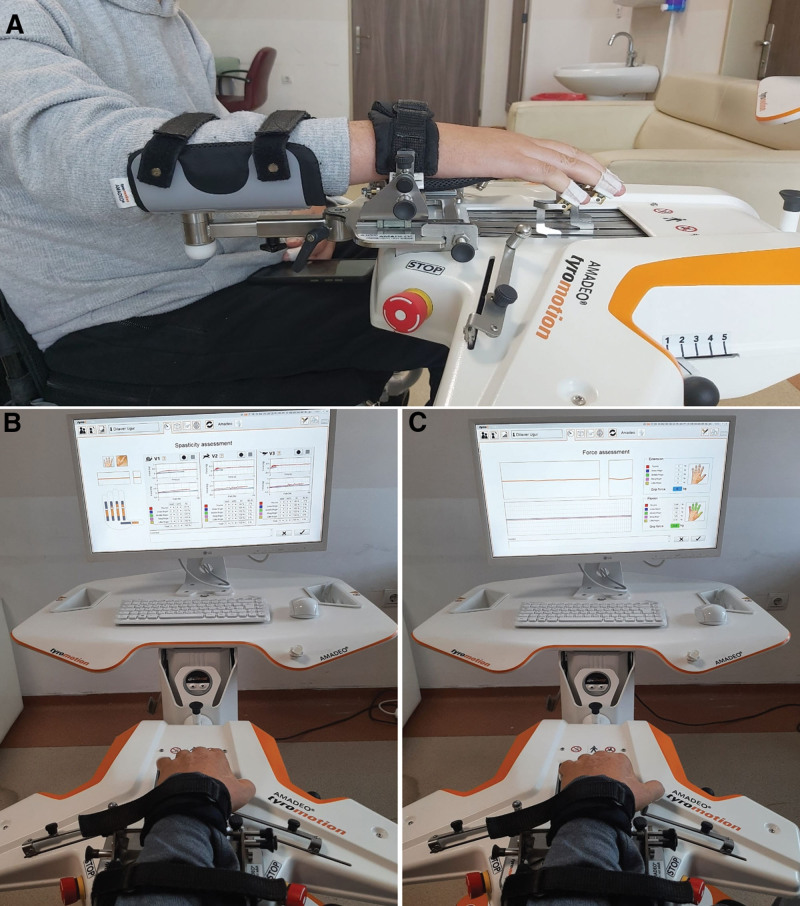
Evaluation position of participants with Amadeo hand finger robotic rehabilitation system.

For robotic evaluation, the patients were seated comfortably with their backs supported, and their affected extremities were placed on the robotic device. Elastic bands and tiny magnets placed on the finger pulp are used to connect the fingers of the user to the robot. There is padded support on the wrist that prevents the user from rotating the arm. The device supports the forearm. The wrist and forearm were secured to the support with a Velcro strap.^[17]^ Before the test, the investigator warned the participants about the association to relax their fingers. Spasticity (three repetitions) and muscle strength (three repetitions) were evaluated during the tests to prevent the onset of spasticity due to the maximum contractions that may occur during the evaluation. The same investigator assessed each participant in 2 sessions using the passive ROM recorded in the initial assessment (Fig. 2).

**Figure 3.**
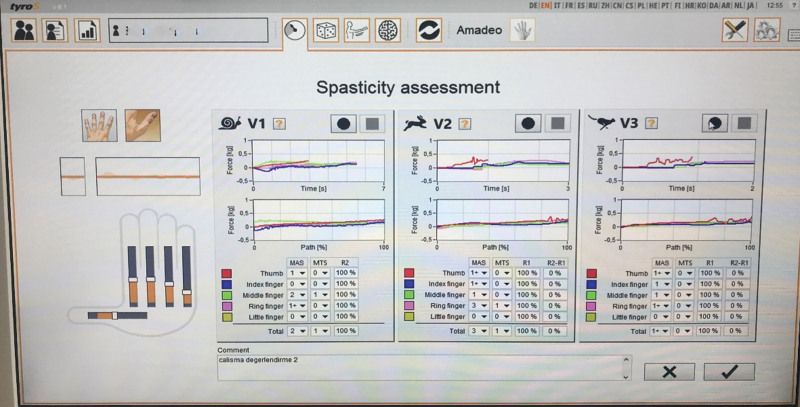
Spasticity evaluation results on Amadeo hand finger robotic rehabilitation system.

For spasticity evaluation, finger sliders moved each finger to their corresponding starting position, as measured during the baseline passive range of motion (ROM) evaluation. Next, the fingers were moved through the entire passive ROM at 3 different speeds: V1 (slow), V2 (medium), and V3 (fast). Velocities are configured such that all fingers initiate and reach their destination simultaneously (i.e., the finger with the shortest ROM moves more slowly than that with the largest ROM)^[12]^ (Fig. 3).

**Figure 4.**
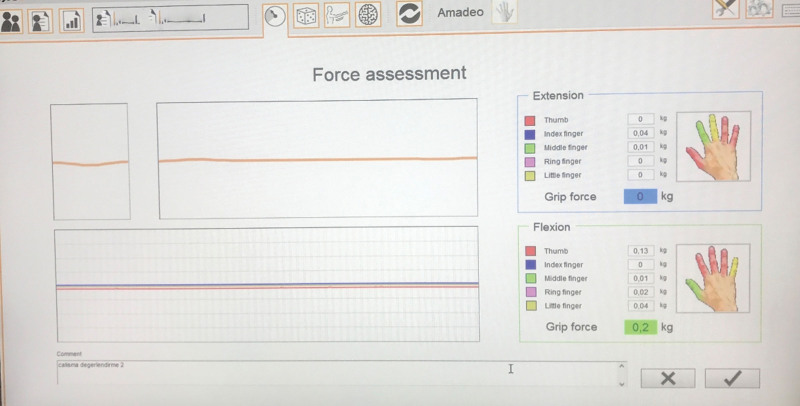
Hand finger strength evaluation results on Amadeo hand finger robotic rehabilitation system.

During hand strength measurements, the participants were instructed to flex their fingers with their last strength for flexion. For extension, a command was provided to open the fingers. The device recorded each finger’s individual and total force measurements in kilograms. Measurements were repeated thrice, and the mean values of the 3 measurements were recorded^[12]^ (Fig. 4).

### 2.5. Sample size calculation

According to the intra-class correlation coefficient (ICC) analysis for test and retest reliability with the G Power package program, the total number of individuals included in the study was a minimum of 158 for alpha 0.05 and beta 0.20 (80% power).

### 2.6. Statistical analysis

Data were analyzed using the IBM SPSS V23 software. The normality of the distribution was checked using the Shapiro–Wilk test. The Mann–Whitney *U* test was used to compare groups for variables that did not follow a normal distribution. Categorical data were compared between groups using Fisher exact test and Pearson’s chi-square test, and multiple comparisons were adjusted using Bonferroni correction. Spearman’s rank correlation coefficient was used to evaluate the relationships between variables that did not follow a normal distribution. Agreement between the 2 assessments was evaluated using the ICC. Descriptive statistics, such as frequency (percentage), mean ± standard deviation, and median (minimum-maximum), were used for categorical and quantitative variables. Statistical significance was set at *P* < .05.

## 3. Results

A total of 161 participants, including 107 patients with hemiplegia and 54 healthy controls, met the inclusion criteria for the study. The mean age of the participants was 62.84 ± 8.06 (40–83), 49.1% (n = 79) were male, and 50.9% were female (n = 82). There was no statistically significant difference between the median ages of the groups (*P* = .07). At the same time, the median age in the patient group was 65 years, and 63 years in the healthy group. The median time after stroke in the patient group was 8 (2–97) months. The affected extremities were on the right side in 45.8% (n = 49) and the left side in 54.2% (n = 58). There was no difference between the patient and control groups in terms of sex, occupation, or dominant hand distribution (*P* > .05) (Table 1).

**Table 1:** Comparison of demographic and clinical variables of patients and healthy controls.

	Groups		Total n (%)	*t*	*P* value
	Patients n (%)	Healthy n (%)			
Sex					
Female	51 (47.7)	28 (51.9)	79 (49.1)	0,252	.616*
Male	56 (52.3)	26 (48.1)	82 (50.9)		
Occupation					
Housewife	47 (43.9)	25 (46.3)	72 (44.7)	5829	.120*
Employee	10 (9.3)	6 (11.1)	16 (9.9)		
Retired	48 (44.9)	18 (33.3)	66 (41)		
Officer	2 (1.9)	5 (9.3)	7 (4.3)		
Dominant hand					
Right	102 (95.3)	51 (94.4)	153 (95)	–	1000**
Left	5 (4.7)	3 (5.6)	8 (5)		

*t* = test statistics; a and b = there is no difference between groups with the same letter for each line.

* Pearson Chi-square test.

†Fisher exact test.

### 3.1. Discriminant ability

The ability of the robotic indices to discriminate patients with stroke from healthy subjects was evaluated. A statistically significant difference was found between the median values of MAS (both clinical and robotic evaluation results) in the patients and healthy subjects (*P* < .01; Table 2). A statistically significant difference was found in both the mean values of hand flexor and extensor strengths measured with the robotic device between the groups (*P* < .01; Table 3).

**Table 2:** Comparison of test-retest results of the clinical and robotic evaluation of finger flexor MAS stages by groups.

	Clinical evaluation				Robotic evaluation			
	Patients	Healthy	*t*	*P* value	Patients	Healthy	*t*	*P* value
	Median (Min–Max)	Median (Min–Max)			Median (Min–Max)	Median (Min–Max)		
MAS-V1 test	1 (0–4)	0 (0–0)	4347	<.001	1 (0–4)	0 (0–1)	4206	<.001
MAS-V2 test	1 (0–4)	0 (0–0)	4374	<.001	1 (0–4)	0 (0–1)	4194	<.001
MAS-V3 test	1 (0–4)	0 (0–0)	4428	<.001	1 (0–4)	0 (0–1)	4281	<.001
MAS-V1 retest	1 (0–4)	0 (0–0)	4428	<.001	1 (0–4)	0 (0–1)	4441	<.001
MAS-V2 retest	1 (0–4)	0 (0–0)	4428	<.001	1 (0–4)	0 (0–1)	4413	<.001
MAS-V3 retest	1 (0–4)	0 (0–0)	4482	<.001	1 (0–4)	0 (0–1)	4315	<.001

MAS = Modified Ashworth Scale, Min-Max = Minimum-Maximum, *t* = test statistics.

*Mann–Whitney *U* test.

**Table 3:** Comparison of robotic measurements of hand strength by groups.

	Groups		Total Mean ± SD	*t*	*P* value*
	Patients Mean ± SD	Healthy Mean ± SD			
Hand flex (kg)	5.01 ± 2.58	8.55 ± 1.7	6.2 ± 2.86	792	<.001
Hand ext (kg)	1.03 ± 1.1	2.94 ± 0.52	1.67 ± 1.31	452	<.001

Mean ± standard deviation.

Hand ext = Hand extension strength, Hand flex = Hand flexion strength, t = test statistics.

* Mann–Whitney *U* test.

### 3.2. Test-retest reliability

Statistically, excellent agreement was obtained between the clinical test and retest results of the MAS in the patient group (ICC = .98–.99; *P* < .01). In addition, excellent agreement was obtained between the robotic device test-retest results of the MAS in the patient group (ICC = .98–.99; *P* < .01) (Table 4).

**Table 4:** Analysis of the agreement between test-retest assessments of finger flexor MAS stages in patients.

	ICC (95% CI)	*P* value
Clinical evaluation		
MAS-V1test – MAS-V1 retest	0.989 (0.985–0.993)	<.001
MAS-V2 test – MAS-V2 retest	0.985 (0.979–0.99)	<.001
MAS-V3 test – MAS-V3 retest	0.987 (0.981–0.991)	<.001
Robotic evaluation		
MAS-V1test – MAS-V1 retest	0.993 (0.99–0.995)	<.001
MAS-V2 test – MAS-V2 retest	0.982 (0.973–0.988)	<.001
MAS-V3 test – MAS-V3 retest	0.993 (0.989–0.995)	<.001

ICC = intraclass correlation coefficient, MAS = Modified Ashworth Scale.

### 3.3. Concurrent validity

There was a statistically significant positive correlation between the MAS scores of the clinical and robotic devices (*r* = .72; *P* < .01). In addition, there was a statistically significant positive correlation between the hand flexor strength values measured with the robotic device, Jamar grip and pinch, and FMA-Hand scores (*P* < .01). There was a statistically significant negative correlation between MAS score and other clinical evaluations (*P* < .01) (Table 5).

**Table 5:** Analysis of the relationship between variables in patients.

A.	MAS-V3 (rob)	Hand flex (kg)	Hand ext (kg)	Jamar grasp	Jamar pinch	FMA Hand
MAS-V3 (cl)	0.724**	−0.621**	−0.756**	−0.721**	−0.729**	−0.756**
MAS-V3 (rob)	1	−0.511**	−0.626**	−0.624**	−0.626**	−0.622**
Hand flex (kg)	B.	1	0.740**	0.694**	0.698**	0.717**
Hand ext (kg)	C.		1	0.729**	0.744**	0.793**
Jamar grasp	D.	E.	F.	1	0.941**	0.739**
Jamar pinch	G.	H.	I.		1	0.721**

cl = clinical evaluation, FMA-Hand = Fugl-Meyer Assessment for upper extremity hand rating subscale, Hand ext = Hand extension strength, Hand flex = Hand flexion strength, MAS = Modified Ashworth Scale, rob = robotic evaluation.

** *P* < .001 *P* value is the result of Spearman correlation.

## 4. Discussion

This study examined the validity and reliability of spasticity and strength measurements using the Amadeo hand finger robotic rehabilitation system in patients with stroke. Our findings show that robotic measurements are reliable and compatible with clinical scales.

Centen et al utilized a robotic exoskeleton to evaluate spasticity at the elbow and reported within-class correlations ranging from 0.66 to 0.95 on mechanical measurements.^[18]^ In a pilot study evaluating the spasticity of 5 patients before and after botulinum toxin administration using an upper extremity robotic device, it was concluded that an exoskeleton robotic device named the NEEM could show even small changes in spasticity.^[10]^ Dehem et al reported that spasticity measurements of 12 stroke patients using the upper extremity robotic device ReaPlan after the motor block procedure correlated with MAS measurements.^[9]^ To the best of our knowledge, only one study has evaluated the spasticity level of finger flexors using a robotic device; Amadeo was used in this study.^[12]^ Germanotta et al reported differences in MAS scores conducted with Amadeo between patients and healthy subjects. Additionally, the reliability of the measurements was poor and did not show a significant correlation with the clinical scale.^[12]^ In contrast to this study, we found that the measurements were reliable and correlated with the clinical scales. Germanotta et al attributed the weak reliability of the results to the low MAS values in the sample and the small variability of MAS in the sample. Germanotta et al included patients in the subacute period (<6 months after stroke).^[12]^ Unlike the study by Germanotta et al, we included patients in the chronic phase. We believe that we obtained a reliable result with a wider MAS distribution of the patients in our study.

Finger grip strength is associated with motor function and activities of daily living.^[19]^ Studies have suggested that muscle weakness has a greater effect on grasping strategies than spasticity.^[20]^ Therefore, the evaluation and rehabilitation of finger strength are as important as those of spasticity. ICCs were found between 0.85 and 0.99 in studies investigating the test-retest reproducibility of the Jamar dynamometer, which is frequently used in the evaluation of hand grip strength in stroke patients.^[21–23]^ For this reason, we evaluated the correlation of our robotic muscle strength measurements with Jamar dynamometer results and the Fugl-Meyer Assessment, the most widely used method to evaluate motor function in stroke patients.^[15]^ A strong positive correlation was observed between the Jamar grip strength measurements and FMA-Hand scores and the finger flexor strength values obtained with the robotic device. Germanotta et al evaluated strength using Modified Research Council (MRC) measurements and found that Amadeo hand strength measurements were in excellent agreement with MRC.^[12]^ In addition, in our study, a significant difference was found between the mean strength of the finger flexors and extensors measured with Amadeo in the patient and healthy groups. All these results show that finger strength measurements measured with Amadeo can distinguish patients from healthy individuals and are compatible with clinical scales.

Dynamometers measuring grip strength were used to measure hand strength. However, as it is known, it is difficult to evaluate muscle strength in stroke patients, especially in patients who do not have hand grip function in the early stages. Although grip is known to be an important function, testing the muscle strength and voluntary flexion movement that will create the grip provides the opportunity to observe the patient in more detail in the early stages of rehabilitation. Robotic devices can measure patients’ active ROM percentages and provide measurements of isolated finger flexion-extension strength. Our study showed that hand flexion strength measured using robotic devices correlated with dynamometer measurements. It was also compatible with the Fugl-Meyer Assessment Hand subscale, which evaluates motor function. In this context, we believe that mechanical muscle strength and spasticity measurements can provide fast, easy-to-apply, reliable, and detailed measurements during the hand rehabilitation process of stroke patients.

The fact that robotic devices, which have begun to be used in treatment practice, can also be used in the evaluation of patients may provide ease of application. In addition, it will provide a chance to avoid the problem of different interpretations between evaluators, which may arise from personal experience and skills. Robotic measurements of spasticity and finger strength are reliable and compatible with clinical scales. Our results will contribute to the literature because there are limited studies in the literature that evaluate finger spasticity and strength using robotic devices. Further studies are needed to determine the current reliability of the measurements of various robotic devices used in rehabilitation practice.

## 5. Limitations

A limitation of this study is that we should have examined the inter-rater reliability. However, this limitation does not significantly affect our results because robotic evaluation is not dependent on the practitioner, and the reliability of manual evaluations has been studied previously.

## 6. Conclusion

Hand finger spasticity and strength measurements of the Amadeo hand-finger robotic rehabilitation system are valid, reliable, and clinically correlated in stroke patients. Robotic spasticity and strength measurements can be used in clinical evaluations to guide treatment plans.

## Author contributions

**Conceptualization:** Sevda Adar, Ali Demircan, Ali İzzet Akçin, Ümit Dündar, Hasan Toktaş, Hilal Yeşil, Selma Eroğlu, Nuran Eyvaz, Ersin Beştaş, Cansu Köseoğlu Toksoy.

**Data curation:** Sevda Adar, Ali Demircan, Ali İzzet Akçin, Nuran Eyvaz, Cansu Köseoğlu Toksoy.

**Formal analysis:** Sevda Adar, Hilal Yeşil, Selma Eroğlu, Nuran Eyvaz.

**Investigation:** Sevda Adar, Ali Demircan, Ali İzzet Akçin, Ümit Dündar, Hasan Toktaş, Hilal Yeşil, Selma Eroğlu, Nuran Eyvaz, Ersin Beştaş, Cansu Köseoğlu Toksoy.

**Methodology:** Sevda Adar, Ümit Dündar, Hasan Toktaş.

**Project administration:** Sevda Adar, Ali İzzet Akçin.

**Resources:** Sevda Adar, Ali Demircan, Ali İzzet Akçin, Ümit Dündar, Hasan Toktaş, Hilal Yeşil, Selma Eroğlu, Nuran Eyvaz, Ersin Beştaş, Cansu Köseoğlu Toksoy.

**Software:** Sevda Adar.

**Validation:** Sevda Adar, Hilal Yeşil, Selma Eroğlu.

**Visualization:** Sevda Adar, Ali Demircan, Ali İzzet Akçin.

**Writing – original draft:** Sevda Adar.

**Writing – review & editing:** Sevda Adar, Ali Demircan, Ali İzzet Akçin, Ümit Dündar, Hasan Toktaş, Hilal Yeşil, Selma Eroğlu, Nuran Eyvaz, Ersin Beştaş, Cansu Köseoğlu Toksoy.
